# The Efficacy of Infliximab Monotherapy versus Infliximab-Azathioprine Sequential Treatment in Crohn's Disease: Experience from a Tertiary Medical Center in China

**DOI:** 10.1155/2016/8648307

**Published:** 2016-11-08

**Authors:** Tianyu Zhang, Zhengting Wang, Rong Fan, Maochen Zhang, Yun Lin, Liwen Hong, Xiaolin Zhou, Shurong Hu, Mengmeng Cheng, Jie Zhong

**Affiliations:** Department of Gastroenterology, Ruijin Hospital, Shanghai Jiaotong University School of Medicine, Shanghai, China

## Abstract

*Objective.* To evaluate the efficacy of infliximab (IFX) monotherapy versus infliximab-azathioprine sequential treatment in Crohn's disease (CD) patients.* Methods.* Patients newly diagnosed with CD using IFX as induction therapy were enrolled. After 6 times of IFX infusions, they were divided into IFX monotherapy group and IFX-AZA sequential therapy group. Clinical remission rates were assessed at weeks 57, 84, 111, and 138 while endoscopic remission rates were assessed at weeks 84 and 138 to evaluate the efficacy of these two groups.* Results.* A total of seventy-nine patients had accomplished 138-week follow-up. At weeks 84 and 138, the deep remission rate (18/22 and 17/22) of IFX monotherapy group was significantly higher compared to IFX-AZA sequential therapy group (26/57 and 21/57) (*P* = 0.004 and 0.001, resp.). Similar findings were found in complete endoscopic remission rate. The clinical remission rates of IFX monotherapy group were similar to that of IFX-AZA sequential therapy group (*P* > 0.05). At weeks 84 and 138, the endoscopic remission rate and the endoscopic improvement rate between these two groups displayed no significant difference (*P* > 0.05).* Conclusion.* IFX monotherapy provides higher deep remission rate compared with IFX-AZA sequential therapy in two-year maintenance therapy. For patients who could not receive prolonged IFX therapy, IFX-AZA sequential therapy is acceptable, though long-term efficacy remains to be seen.

## 1. Introduction

Crohn's disease (CD) is a chronic granulomatous inflammatory disorder with an involvement of various skipping segments of gastrointestinal tract [1]. The past two decades have seen a great increase in the incidence of CD, with 3-fold increase in China. These changes may be due to an increased contact with the West, westernization of diet, increased use of antibiotics, improved hygiene, vaccinations, or changes in the gut microbiota [2, 3].

Treatment of CD includes two phases, induction of remission and maintenance of remission [4, 5]. Conventionally, treatment escalation with the use of increasingly potent immunomodulator has been applied in a stepwise fashion, termed as “step-up” strategy. With the introduction and wide use of anti-TNF*α* agents in the past two decades, the natural course of CD patients has been obviously changed, with reduction of clinical relapse, future surgery, and hospitalization. Consequently, a more aggressive “top-down” strategy has been advocated in recent years, supported by evidence that early use of anti-TNF*α* monotherapy or combined therapy with AZA induces higher remission rates in CD [6–8]. However, medication period for CD patients is fairly long as recommendation regarding treatment cessation is not at hand [9]. There is a growing concern that long-term combined therapy may bring increasing risk of serious infections [10], lymphoproliferative disorders [11], and nonmelanoma skin cancer [12]. Also, there is a cost-benefit concern regarding the high hygiene burden of CD treatment in our country [13].

In such cases, our aim of this study was to evaluate the efficacy of IFX monotherapy versus IFX-AZA sequential therapy in CD patients.

## 2. Methods

### 2.1. Patients Enrolled

The institutional review board approved our study and informed consent was obtained from all patients. This was an open-label, observational study. All of the newly diagnosed CD patients were screened in our study, with a Crohn's Disease Activity Index (CDAI) > 150 in Department of Gastroenterology, Ruijin Hospital, from August 2012 to March 2014. After a thorough evaluation of their high risk factors, patients chose the treatment strategy according to the recommendation of doctors and their own choice. Patients who received IFX monotherapy and acquired remission after 6 times' infusions were eligible for our final cohort.

### 2.2. Inclusion and Exclusion Criteria

Inclusion criteria include the following: (1) age between 18 and 70 years; (2) CDAI < 150; and (3) acquired remission by IFX monotherapy at a dose of 5 mg/kg after scheduled 6 infusions. Exclusion criteria were as follows: (1) patients who had fibrostenosis or phlegmon or any other complications that had contraindications of biologics or immunosuppressants; (2) current knowledge of tuberculosis, HIV, HBV, or HCV infections; (3) current or intended pregnancy; (4) other severe renal, hepatic, or heart disease; and (5) patients who did not give informed consent.

### 2.3. Study Design

Before the study, all patients underwent thorough clinical assessment. Basic information included the patient's age, gender, duration of symptom, smoking history, and CD-related surgery. Montreal classification of each patient was recorded including age at diagnosis, location, disease behavior, and perianal disease. Colonoscopy or double-balloon enteroscopy was performed to detect the luminal lesions and calculate Crohn's Disease Endoscopic Index of Severity (CDEIS) while CTE or MRE was performed to have knowledge of extraintestinal lesions and complications. Baseline data including CRP, ESR, WBC, CDAI, and CDEIS were recorded. Routine hematological and biochemical tests were performed to exclude the potential contraindications of biologics and immunosuppressants. All patients received induction therapy with infliximab (Remicade, Xi'an-Janssen, China) at a dose of 5 mg/kg intravenously at weeks 0, 2, 6, 14, 22, and 30. Then patients who acquired clinical remission were asked to choose the maintenance therapy with IFX or AZA according to their own economic status. The IFX monotherapy group continued a scheduled IFX infusion from week 38 at an 8-week interval. The IFX-AZA sequential therapy group received azathioprine (Imuran, GlaxoSmithKline, Brentford, Middlesex, United Kingdom) from week 31 onwards, starting with an initial dose of 50 mg followed by increasing 25 mg biweekly to the maximally tolerated dose according to the white blood cell and liver function test.

### 2.4. Efficacy Assessment

The primary endpoints of this study were deep remission rates as defined by clinical remission plus mucosal healing (MH) at weeks 84 and 138. The secondary endpoints included the clinical remission rate at weeks 57, 84, 111, and 138 and endoscopic remission at weeks 84 and 138. Clinical remission was defined as Crohn's Disease Activity Index (CDAI) < 150 while complete MH was defined as absence of active mucosal ulcerations. All patients had CTE or MRE to determine the location of lesions; then those who were exclusively small bowel involved had underwent double-balloon enteroscopy (DBE) performed by Professor Jie Zhong through oral (antegrade) or rectal (retrograde) approaches to evaluate at least 1 m of intestinal lesion segment. Endoscopic remission was defined as Crohn's Disease Endoscopic Index of Severity (CDEIS) < 6. Endoscopic improvement was defined as a decrease in CDEIS score of >5. Complete endoscopic remission was defined as CDEIS score < 3. The CDEIS was independently graded by 2 endoscopists without being aware of patient's clinical status and average value was taken. Patients who received a drug not allowed by the protocol, who had CD-related surgery, who had underwent severe medical side-effect, or who discontinued follow-up due to lack of efficacy or loss of response were judged as treatment failure, irrespective of CDAI score.

### 2.5. Statistical Analysis

SPSS 21.0 (IBM, Chicago, Illinois, USA) was used to perform further analysis. Measurement variables are expressed as mean ± standard deviation (SD) for data conformed to normal distribution. Comparison of measurement variables was performed using Student's* t*-test depending on data distribution. Qualitative variables were described as *n* and percentage and comparison of ratios was tested by *χ* ^2^ test. *P* < 0.05 was considered statistically significant.

## 3. Results

### 3.1. Baseline Characteristics

**Figure 1:**
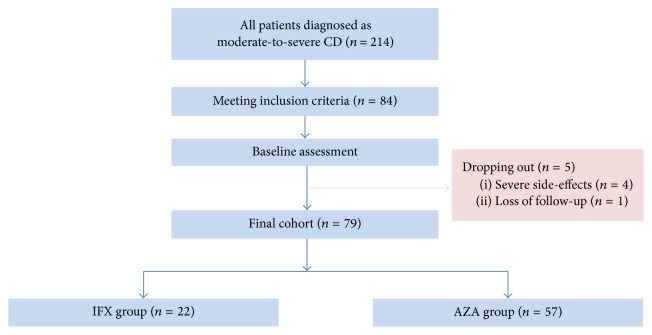
Flowchart of this observational study. CD, Crohn's disease; CDAI, Crohn's Disease Activity Index; CDEIS, Crohn's Disease Endoscopic Index of Severity; IFX, infliximab; and AZA, azathioprine.

From August 2012 to March 2014, a total of 214 patients were newly diagnosed as moderate-to-severe active Crohn's disease. 84 patients received IFX as induction therapy. 79 patients completed our open-label, observational study after two years of follow-up, with 5 dropping out because of severe side-effects (leucopenia 2, acute pancreatitis 1, and infusion reaction 1) and loss of follow-up (1). Flowchart of this study was displayed in Figure 1. Among the final cohort, 22 patients chose IFX as maintenance therapy while 57 choose AZA. No significant difference was found between IFX monotherapy group and IFX-AZA sequential therapy group regarding gender, duration of symptoms, and age of onset (*P* > 0.05) (Table 1). Location and behavior of disease were similar between the two groups (*P* > 0.05) (Table 1). The incidence of perianal disease rate was not significantly different between IFX monotherapy group and IFX-AZA sequential therapy group (*P* > 0.05) (Table 1). Compared with IFX monotherapy group, the baseline CDAI score and CDEIS score of IFX-AZA sequential therapy group were also of no significant difference (*P* > 0.05) (Table 1). By week 30, 44/79 (55.7%) patients acquired deep remission in total. The deep remission rate between IFX monotherapy group and IFX-AZA sequential therapy group was not significantly different (*P* = 0.706). Complete endoscopic remission rate was similar between IFX monotherapy group and IFX-AZA sequential therapy group at week 30 (59.1% versus 54.4%). The endoscopic remission rate, characterized by CDEIS < 6, showed no significant difference at week 30 (Table 1).

### 3.2. Efficacy Evaluation

Patients who chose IFX as maintenance therapy reached significantly higher deep remission rate (18/22, 81.8%) than those who chose AZA (26/57, 45.6%) at week 84 (*P* = 0.004). Similar results were found at week 138, with 17/22 (77.3%) of IFX monotherapy group and 21/57 (36.8%) of IFX-AZA sequential therapy (*P* = 0.001) (Table 2).

In maintenance of remission phases (weeks 57, 84, 111, and 138), the clinical remission rate of IFX monotherapy group was slightly higher compared to IFX-AZA sequential therapy group, but with no significant difference (*P* = 0.871, 0.830, 0.999, and 0.665, resp.) (Table 2).

In terms of endoscopic response, as we continued IFX therapy in maintaining remission, the complete endoscopic remission rate at weeks 84 and 138 improved compared with AZA as maintenance regime, which had reached significant difference (*P* = 0.012 and 0.002, resp.). The endoscopic remission rate, characterized by CDEIS < 6, showed no significant difference at weeks 84 and 138 between these two groups (*P* = 0.365, 0.257, and 0.665, resp.). Over 90% of patients in our study had reached and maintained endoscopic improvement during over 2 years of study time, with no significant difference between the two groups (*P* = 0.999).

## 4. Discussion

Combination therapy with IFX and IMM has been proven to be more effective in CD maintenance therapy than either treatment alone [14]. However, the optimal duration of combination therapy is debated, especially in the patients with long symptom-free phase. Moreover, there is a recognized risk of non-Hodgkin's lymphoma (NHL) especially in patients receiving combination therapy [11]. Particular concern had been raised as the recent reported cluster of hepatosplenic T-cell lymphoma in CD patients occurred, with 17 cases in total [15]. All except one were receiving biologics and IMM combined therapy, and the prognosis of these patients was fatal. Also, we could not afford to ignore opportunistic infection of combined therapy. Toruner et al. demonstrated that the respective use of corticosteroids, IMM, or IFX conferred a 3-fold increased risk of developing an opportunistic infection while it increased 15-fold when two or more therapies are used in combination [16]. On the other hand, the cost of IFX therapy is high as it has not been included in assigned drugs of medical insurance in most areas of developing countries like China. Consequently, a large proportion of patients could not afford long-term use of combined therapy. Thus, it is of great importance and necessity to formulate an optimal treatment strategy to balance efficacy, side-effects, and cost-effectiveness in CD treatment. Sufficiency but not excess should always be the goal to treat. Thus, in our study, we evaluated the efficacy of IFX monotherapy or IFX-AZA sequential therapy treatment.

There have been several studies focused on discontinuation of combined therapy in CD treatment. Van Assche et al. discovered that continuation of IMM beyond 6 months offered no clear benefit over scheduled IFX monotherapy [17]. Oussalah et al. showed that AZA withdrawal was associated with high risk of relapse in patients with a duration of combined therapy of <27 months and/or the presence of biological inflammation [18]. Louis et al. found that approximately 50% of patients with CD who were treated for at least 1 year with IFX and IMM experienced a relapse within 1 year after discontinuation of IFX [19]. A multidisciplinary European expert panel observed that IFX withdrawal was considered appropriate after two years of clinical remission in case of combined therapy [20]. A recently published study by Ampuero et al. found that a subgroup of Crohn's disease patients treated with combination therapy can be identified (C-reactive protein < 5 mg/L, endoscopic remission, and older age at Crohn's disease diagnosis) who would continue in remission despite cessation of the biological (expensive) component of the combination therapy [21]. Our study focused on comparing IFX monotherapy and IFX-AZA sequential therapy treatment in CD which had rarely been studied before in well-designed prospective study. The results of our study showed that, after 6 times of scheduled infusions of IFX and sequential AZA treatment, CD patients acquired high clinical remission rate (78.9%–94.7%) at each time point for 2-year duration. The remission rate between the IFX monotherapy group and IFX-AZA sequential therapy group had no significant difference. So IFX-based induction therapy followed by 2 years of AZA as maintenance therapy is acceptable for CD patients.

In recent years, deep remission has become an evolving therapeutic target in CD, characterized by clinical remission plus mucosal healing [22]. More physicians focus on endoscopic response instead of clinical symptoms alone [23]. Schnitzler et al. found that both MH (0 ulcers) and partial MH (defined as endoscopic improvement but still with some ulcers present) reached by IFX maintenance therapy improved the long-term outcome in patients with CD [24]. Furthermore, a post hoc analysis of SONIC demonstrated that CD patients that achieved MH or endoscopic response defined as CDEIS decrease ≥50% from baseline at week 26 of treatment were most likely to be in corticosteroid-free remission at week 50 [25]. Thus, in our study, we assessed the rate of deep remission, complete endoscopic remission, endoscopic remission, and endoscopic improvement of IFX monotherapy group and IFX-AZA sequential therapy group. At week 84 and week 138, the IFX monotherapy group reached significantly higher deep remission and complete endoscopic remission rate compared to IFX-AZA sequential therapy group. But, in endoscopic remission and endoscopic improvement rate, the two groups displayed no significant difference during 138-week follow-up. Further study is needed to evaluate longer time remission rate between these two groups.

There are some limitations of our study. Firstly, the cohort of our study was limited, especially patients in IFX monotherapy group. This was mainly due to the economic status of Chinese patients. In the future, as biological agents are being added to medical insurance, more patients may have the opportunity of using biologics as maintenance therapy. Secondly, we did not assign the patients to more specific subgroup in order to provide treatment recommendation according to baseline risk factors of each patient.

In conclusion, IFX monotherapy provides higher deep remission rate compared with IFX-AZA sequential therapy. For patients who could not receive prolonged IFX therapy, IFX-AZA sequential therapy is acceptable, though long-term efficacy remains to be seen.

## Figures and Tables

**Table 1:** Baseline characteristics of our study.

Variables	IFX monotherapy group (*N* = 22)	IFX-AZA sequential therapy group (*N* = 57)	All (*N* = 79)	*P* value
Male sex, *n* (%)	16 (72.7)	38 (66.7)	54 (68.4)	0.604
Duration of symptoms (week)	27.1 ± 13.5	28.3 ± 14.1	28.0 ± 13.9	0.733
Age of onset (yr)	26.9 ± 9.8	24.2 ± 8.2	25.0 ± 8.7	0.217
Location of lesions, *n* (%)				0.829
Ileum	6 (27.3)	20 (35.1)	26 (32.9)	
Colon	3 (13.6)	6 (10.5)	9 (11.4)	
Ileocolon	12 (54.5)	30 (52.6)	42 (53.2)	
Upper GI tract	1 (4.5)	1 (1.8)	2 (2.5)	
Behavior of disease, *n* (%)				0.252
Nonstenosis, nonfistula	14 (63.6)	46 (80.7)	60 (75.9)	
Stenosis	4 (18.2)	7 (12.3)	11 (13.9)	
Fistula	4 (18.2)	4 (7.0)	8 (10.1)	
Perianal disease, *n* (%)	9 (40.9)	20 (35.1)	29 (36.7)	0.630
CDAI	344.9 ± 51.0	331.8 ± 61.6	334.9 ± 58.7	0.457
CDEIS	14.2 ± 3.8	14.1 ± 3.9	14.1 ± 3.9	0.964
Deep remission at week 30	13 (59.1)	31 (54.4)	44 (55.7)	0.706
Complete endoscopic remission at week 30	13 (59.1)	31 (54.4)	44 (55.7)	0.706
Endoscopic remission at week 30	19 (86.4)	42 (73.7)	61 (77.2)	0.365
Endoscopic improvement at week 30	21 (95.5)	54 (94.7)	75 (94.9)	0.999

**Table 2:** Efficacy assessment of IFX or AZA monotherapy in maintaining therapy of Crohn's disease, *n* (%).

Efficacy assessment	IFX monotherapy group (*N* = 22)	IFX-AZA sequential therapy group (*N* = 57)	All (*N* = 79)	*P* value
Deep remission at week 84	18 (81.8)	26 (45.6)	44 (55.7)	0.004
Deep remission at week 138	17 (77.3)	21 (36.8)	38 (48.1)	0.001
Clinical remission at week 57	21 (95.5)	52 (91.2)	73 (92.4)	0.871
Clinical remission at week 84	20 (90.9)	49 (86.0)	69 (87.3)	0.830
Clinical remission at week 111	19 (86.4)	48 (84.2)	67 (84.8)	0.999
Clinical remission at week 138	19 (86.4)	45 (78.9)	64 (81.0)	0.665
Complete endoscopic remission at week 84	18 (81.8)	27 (47.4)	45 (57.0)	0.012
Complete endoscopic remission at week 138	17 (77.3)	22 (38.6)	39 (49.4)	0.002
Endoscopic remission at week 84	21 (95.5)	47 (82.5)	68 (86.1)	0.257
Endoscopic remission at week 138	19 (86.4)	45 (78.9)	64 (81.0)	0.665
Endoscopic improvement at week 84	21 (95.5)	53 (93.0)	74 (93.7)	0.999
Endoscopic improvement at week 138	20 (90.9)	52 (91.2)	72 (91.1)	0.999
